# Trans fatty acid intake and risk of type 2 diabetes in the NutriNet-Santé prospective cohort

**DOI:** 10.1093/eurpub/ckac129.694

**Published:** 2022-10-25

**Authors:** G Wendeu-Foyet, A Bellicha, V Chajes, I Huybrechts, C Debras, B Srour, L Sellem, L Fezeu, M Deschasaux-Tanguy, M Touvier

**Affiliations:** 1 Nutritional Epidemiology Research Team, Inserm U1153, Inrae U1125, Bobigny, France; 2 Nutrition and Cancer Research Network, Jouy-en-Josas, France; 3 Director’s Office, International Agency for Research on Cancer, Lyon, France

## Abstract

Type 2 diabetes (T2D) is one of the most common noncommunicable diseases worldwide, with an increasing prevalence and a considerable global health burden. Substantial evidence has linked consumption of trans fatty acids (TFAs) to an increased risk of cardiovascular disease. However, the effects of T2D remain unclear. We aimed to investigate the associations between different types of TFAs (total, ruminant, industrial and corresponding specific isomers) and risk of T2D in the NutriNet-Santé prospective cohort. Overall, 105,551 participants aged 18 years or older from the French NutriNet-Santé cohort (2009-2021) were included (mean age at baseline=42.7y (SD = 14.6y), 79.2% women). Dietary intake data, including usual TFA intake, were collected using repeated 24-hour dietary records (n = 5.7 [SD = 3.1]). Associations between sex-specific quartiles of dietary intake of TFAs and type 2 diabetes risk were assessed using multivariable Cox proportional hazard models adjusted for known risk factors. A total of 969 incident type 2 diabetes cases occured during follow-up. Total TFAs was associated with higher T2D risk (HRfor quartile 4 versus 1=1.38; 95% CI = 1.11-1.73; Ptrend<0.001). This association, specifically observed for industrial TFAs (HR = 1.45; 95% CI = 1.15-1.83; Ptrend<0.001), was mainly driven by elaidic acid (HR = 1.37; 95% CI = 1.09-1.72; Ptrend<0.001) and linolelaidic acid (HR = 1.29; 95% CI = 1.04-1.58; Ptrend=0.07). In contrast, ruminant trans fatty acids were not significantly associated with the risk of T2D. In this large prospective cohort, higher dietary intakes of total and industrial TFAs were associated with increased T2D risk. These findings support WHO's recommendation to eliminate industrially-produced TFAs from the food supply worldwide. As such, consumers should be advised to limit the consumption of food products containing partially hydrogenated oils (main vector of iTFAs) as this, specifically, may contribute to lower the substantial global burden of T2D.

**Key messages:**

• Higher dietary intakes of total and industrial trans fatty acids were associated with increased type 2 diabetes risk.

• Our findings support WHO’s recommendation to eliminate industrially-produced TFAs from the food supply worldwide.

